# Wheat MIXTA-like Transcriptional Activators Positively Regulate Cuticular Wax Accumulation

**DOI:** 10.3390/ijms25126557

**Published:** 2024-06-14

**Authors:** Xiaoyu Wang, Yixian Fu, Xiaofeng Liu, Cheng Chang

**Affiliations:** College of Life Sciences, Qingdao University, Qingdao 266071, China

**Keywords:** wheat, MIXTA-like transcription factor, wax biosynthesis, TaMIXTA1, TaMIXTA1, *TaCER5*, *TaKCS1*

## Abstract

MIXTA-like transcription factors AtMYB16 and AtMYB106 play important roles in the regulation of cuticular wax accumulation in dicot model plant *Arabidopsis thaliana*, but there are very few studies on the MIXTA-like transcription factors in monocot plants. Herein, wheat MIXTA-like transcription factors TaMIXTA1 and TaMIXTA2 were characterized as positive regulators of cuticular wax accumulation. The virus-induced gene silencing experiments showed that knock-down of wheat *TaMIXTA1* and *TaMIXTA2* expressions resulted in the decreased accumulation of leaf cuticular wax, increased leaf water loss rate, and potentiated chlorophyll leaching. Furthermore, three wheat orthologous genes of *ECERIFERUM 5* (*TaCER5-1A*, *1B*, and *1D*) and their function in cuticular wax deposition were reported. The silencing of *TaCER5* by BSMV-VIGS led to reduced loads of leaf cuticular wax and enhanced rates of leaf water loss and chlorophyll leaching, indicating the essential role of the *TaCER5* gene in the deposition of wheat cuticular wax. In addition, we demonstrated that TaMIXTA1 and TaMIXTA2 function as transcriptional activators and could directly stimulate the transcription of wax biosynthesis gene *TaKCS1* and wax deposition gene *TaCER5*. The above results strongly support that wheat MIXTA-Like transcriptional activators TaMIXTA1 and TaMIXTA2 positively regulate cuticular wax accumulation via activating *TaKCS1* and *TaCER5* gene transcription.

## 1. Introduction

The waxy cuticle represents the outmost surface of land plants and covers the nonwoody plant organs such as leaves, stems, flowers, and even underground portions like root tips [1,2]. As one of the most important innovations during plant terrestrialization, the lipophilic cuticle restricts non-stomatal water loss and gas exchanges and shields plant tissues from environmental challenges associated with land colonization, including desiccation, extreme temperatures, and ultraviolet (UV) radiation [3,4,5,6,7,8,9,10,11]. In addition to these protective roles, the cuticle governs plant developmental events such as organ separation and lateral root formation [12,13]. Due to their essential roles in plant development and environmental adaptation, cuticle-associated traits like leaf wax alkane concentration have been selected in breeding efforts for grain yield improvement in the important cereal crop bread wheat (*Triticum aestivum* L.) [14,15,16,17].

As the organic solvent-extractable constituents, wax mixtures impregnate and seal the organic solvent-insoluble cutin matrices in the cuticle [18,19,20,21,22,23,24]. Unlike cutin usually comprising polyesters of oxygenated C16 and C18 fatty acids, wax mixtures are mainly composed of very long-chain (VLC, >C20) fatty acids and their derivatives, including VLC alkanes, VLC primary and secondary alcohols, VLC aldehydes, VLC ketones, and VLC esters [18,19,20,21,22,23,24]. The composition and amounts of cuticular wax not only vary among plant species and organs but also depend on environmental conditions and plant developmental stages [18,19,20,21,22,23,24]. As extensively studied in the dicot model plant *Arabidopsis thaliana*, cuticular wax mixtures are mainly synthesized in the endoplasmic reticulum (ER) of plant epidermal cells via elongation and modification of C16 and C18 fatty acids transported from the plastid [18,19,20,21,22,23,24]. As previously reviewed, C16 and C18 fatty acids are firstly esterified to form acyl-CoAs under the action of long-chain acyl-coenzyme A synthases (LACS), and then they undergo aliphatic chain elongation to generate VLC acyl-CoAs by the fatty acid elongase (FAE) complexes composed of ketoacyl-CoA synthases (KCSs), ketoacyl-CoA reductases (KCRs), hydroxyacyl-CoA dehydratases (HCDs), and enoyl-CoA reductases (ECRs), together with action of the cofactor ECERIFERUM2-LIKE (CER2-LIKE) proteins [25,26,27,28,29,30,31,32,33,34,35,36,37,38,39,40,41]. In the ER, these elongated VLC acyl-CoAs could be further modified into VLC aldehydes, VLC alkanes, VLC secondary alcohols, and VLC ketones via the alkane-forming pathway, or into VLC primary alcohols and esters via the alcohol-forming pathway [42,43,44,45,46,47,48,49,50]. These VLC fatty acids and derivatives are then exported out of the ER, across the plasma membrane (PM), and to the extracellular cuticular regions presumably under the sequential action of the intracellular Golgi and trans-Golgi network (TGN)-trafficking pathways, PM-localized ATP binding cassette (ABC) transporters like ECERIFERUM5 (AtCER5), and the secreted lipid transfer proteins (LTPs) [51,52,53,54,55,56,57,58,59,60,61,62].

As discussed in prior reviews, cuticular wax biosynthesis is tightly governed by a variety of regulators at transcriptional, post-transcriptional, post-translational, and even epigenetic levels [21]. For instance, Arabidopsis APETALA2/ethylene response factor (AP2/ERF) transcription factors SHINE1/WAXINDUCER1 (AtSHN1/WIN1), DEWAX, DEWAX2, WRINKLED4 (AtWRI4), and RELATED TO APETALA 2.4 (AtRAP2.4) and R2R3-type myeloblastosis (MYB) transcription factors AtMYB94 and AtMYB96 govern the transcription of the wax biosynthesis gene and regulate cuticular wax biosynthesis in response to developmental and environmental cues [63,64,65,66,67,68,69,70,71,72,73,74,75,76,77,78]. RNA processing machinery including Arabidopsis RNA exosome components and cofactors AtCER7/RRP45B, AtRST1, and AtRIPR/CER16; SUPERKILLER complex components AtSKI2, AtSKI3, and AtSKI8; and RNA-mediated gene silencing components AtRDR1, AtRDR6, AtSGS3, and AtSDE3 become involved in the post-transcriptional regulation of wax biosynthesis genes like *ECERIFERUM3* (*CER3*), thereby fine-tuning cuticular wax biosynthesis [79,80,81]. Arabidopsis E3 ubiquitin ligases SMALL AND GLOSSY LEAVES1 (AtSAGL1) mediates the polyubiquitination and proteasomal degradation of AtCER3 proteins, thereby governing wax biosynthesis at post-translational levels [82]. In addition, Arabidopsis histone methyltransferase AtSDG8 and histone acetyltransferase AtGCN5 mediate histone modification and regulate wax biosynthesis gene transcription, suggesting that these histone modifiers become involved in the epigenetic control of cuticular wax biosynthesis [83,84]. Although unprecedented progress has been obtained in the study of wax biosynthesis in the dicot model plant *A. thaliana*, regulatory mechanisms underlying cuticular wax biosynthesis in agronomically important crops like bread wheat are poorly understood.

In this research, two MIXTA-like transcription factors TaMIXTA1 and TaMIXTA2 were characterized as positive regulators of wheat cuticular wax accumulation. BSMV-VIGS experiments showed that the knock-down of *TaMIXTA1* and *TaMIXTA2* expression resulted in the decreased accumulation of leaf cuticular wax, increased leaf water loss rate, and potentiated chlorophyll leaching. Furthermore, wheat ortholog genes of *CER5* (*TaCER5-1A*, *1B*, and *1D*) were found to be essential for cuticular wax deposition. The virus-induced gene silencing of *TaCER5* led to the reduced deposition of leaf cuticular wax and enhanced rates of leaf water loss and chlorophyll leaching. Importantly, TaMIXTA1 and TaMIXTA2 proteins exhibit transcriptional activating activity and could directly stimulate the transcription of wax biosynthesis gene *TaKCS1* and wax deposition gene *TaCER5*. This study for the first time elucidates the regulatory role of MIXTA-like proteins on cuticular wax biosynthesis and transport in the agronomically important crop bread wheat.

## 2. Results

### 2.1. Identification of Wheat TaMIXTA1 and TaMIXTA2 Based on Homology with Arabidopsis MIXTA-like Proteins

**Figure 1:**
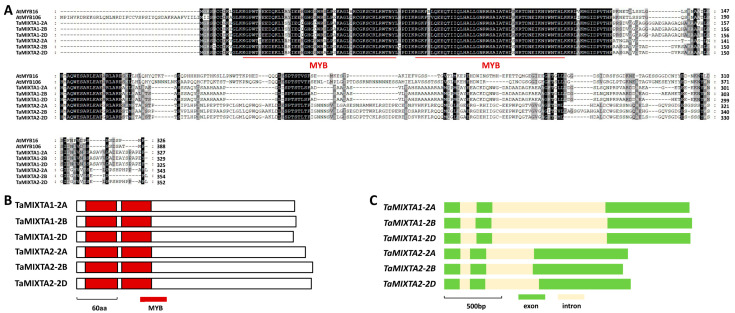
Homology-based identification of wheat MIXTA-like MYB transcription factors. (**A**) Protein sequence comparison of wheat TaMIXTA1, TaMIXTA2, and Arabidopsis MIXTA-like MYB transcription factors AtMYB16 and AtMYB106. Identical residues among 8 protein sequences are shaded in black, while residues conserved in at least 4 of the 8 proteins are shaded in gray. (**B**) Domain structures of wheat TaMIXTA1 and TaMIXTA2 proteins. (**C**) Gene architectures of wheat *TaMIXTA1* and *TaMIXTA2* genes.

Arabidopsis MIXTA-like transcription factors AtMYB16 and AtMYB106 play important roles in the regulation of cuticular wax biosynthesis, but the MIXTA-like proteins in the agronomically important cereal bread wheat remain to be identified. In this study, we employed the Arabidopsis AtMYB16 (At5g15310) and AtMYB106 (At3g01140) as queries to search the reference genome of allohexaploid bread wheat. Wheat MIXTA-like proteins TaMIXTA1 and TaMIXTA2 were identified as the closed homologs of Arabidopsis MIXTA-like transcription factors AtMYB16 and AtMYB106. As shown in Figure 1A, *TaMIXTA1-2A* (*TraesCS2A02G338200*), *TaMIXTA1-2B* (*TraesCS2B02G343800*), and *TaMIXTA1-2D* (*TraesCS2D02G324800*) located on chromosomes 2A, 2B, and 2D of allohexaploid bread wheat are three highly homologous sequences of the *TaMIXTA1* gene. Similarly, *TaMIXTA2-2A* (*TraesCS2A02G552400*), *TaMIXTA2-2B* (*TraesCS2B02G583800*), and *TaMIXTA2-2D* located on wheat chromosomes 2A, 2B, and 2D are three highly homologous sequences of *TaMIXTA2* genes. 

Amino acid sequences of the TaMIXTA1-2A, TaMIXTA1-2B, TaMIXTA1-2D, TaMIXTA2-2A, TaMIXTA2-2B, and TaMIXTA2-2D proteins shared above 46% identities with Arabidopsis AtMYB16 and AtMYB106 proteins (Figure 1A). As depicted in Figure 1B, two MYB motifs appear in the N-terminal parts of the TaMIXTA1-2A, TaMIXTA1-2B, TaMIXTA1-2D, TaMIXTA2-2A, TaMIXTA2-2B, and TaMIXTA2-2D proteins. Gene architecture analysis revealed that three exons and two introns constitute the coding regions of genomic sequences of the *TaMIXTA1-2A*, *TaMIXTA1-2B*, *TaMIXTA1-2D*, *TaMIXTA2-2A*, *TaMIXTA2-2B*, and *TaMIXTA2-2D* genes (Figure 1C).

### 2.2. Wheat-Redundant MIXTA-like Transcription Factors Positively Regulate Cuticular Wax Accumulation

**Figure 2:**
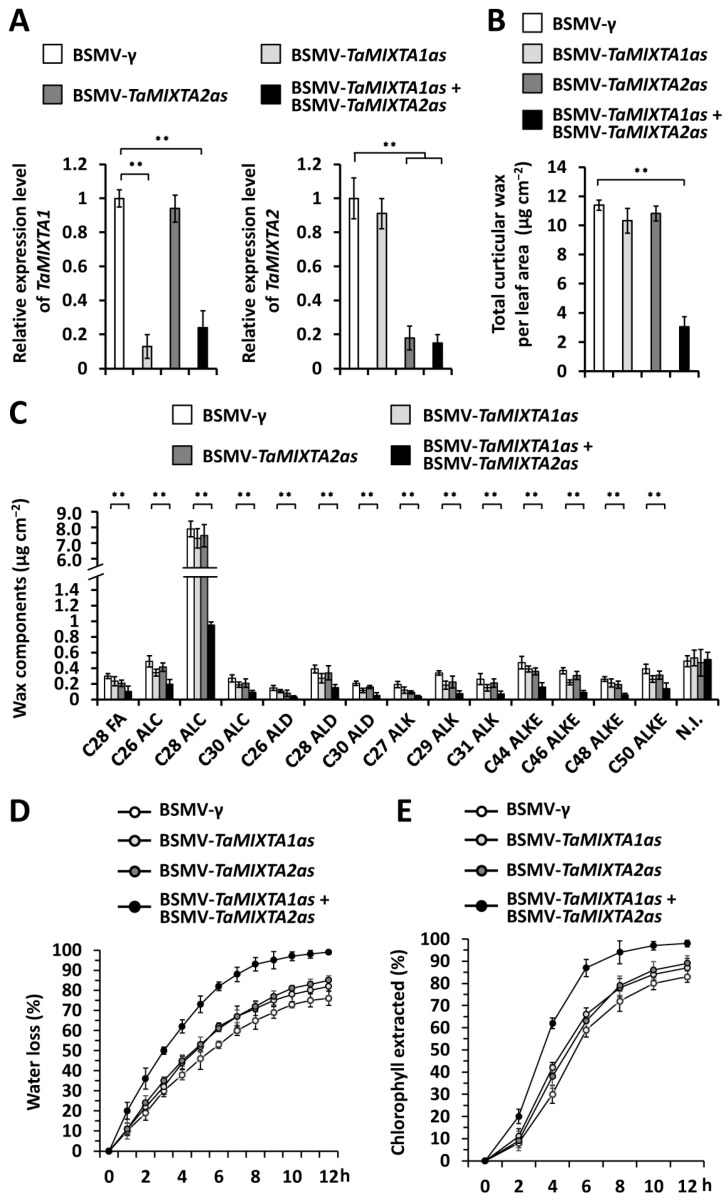
Functional analyses of wheat MIXTA-like genes in cuticular wax accumulation. (**A**) qRT-PCR analysis of *TaMIXTA1* and *TaMIXTA2* expression levels in the wheat leaves silencing *TaMIXTA1* (BSMV-*TaMIXTA1as*), *TaMIXTA2* (BSMV-*TaMIXTA2as*), or co-silencing *TaMIXTA1* and *TaMIXTA2* (BSMV-*TaMIXTA1as* + BSMV-*TaMIXTA2as*). (**B**) Total cuticular wax amounts in the wheat leaves silencing *TaMIXTA1*, *TaMIXTA2*, or co-silencing *TaMIXTA1* and *TaMIXTA2*. (**C**) Amounts of major cuticular wax components in the wheat leaves silencing *TaMIXTA1*, *TaMIXTA2*, or co-silencing *TaMIXTA1* and *TaMIXTA2*. FA, fatty acid; ALC, alcohol; ALD, aldehyde; ALK, alkane; ALKE, alkyl ester; N.I., not identified compound. (**D**) Water loss rates and (**E**) chlorophyll extraction levels analyzed in wheat leaves silencing *TaMIXTA1*, *TaMIXTA2*, or co-silencing *TaMIXTA1* and *TaMIXTA2.* For (**A**–**E**), three biological replicates were statistically analyzed for each treatment, and data are presented as the mean ± SE (Student’s *t*-test, ** *p* < 0.01).

To examine the potential regulation of cuticular wax accumulation by wheat *TaMIXTA1* and *TaMIXTA2* genes, we silenced all endogenous *TaMIXTA1* or *TaMIXTA2* genes by performing the barley stripe mosaic virus-induced gene silencing (BSMV-VIGS) assay in the plants of wheat cultivar Yannong 999. The quantitative reverse transcription-polymerase chain reaction (qRT-PCR) assay demonstrated that the expression levels of *TaMIXTA1* or *TaMIXTA2* genes decreased significantly in wheat leaves silencing *TaMIXTA1*, *TaMIXTA2*, or co-silencing *TaMIXTA1* and *TaMIXTA2* (Figure 2A). Cuticular wax mixtures were then extracted from these wheat leaves and subjected to the gas chromatography–mass spectrometry (GC-MS) analysis. The amount of cuticular wax accumulated on wheat leaves decreased from 11.39 μg cm^−2^ in the BSMV-*γ* infected plants to 3.06 μg cm^−2^ in the wheat plants co-silencing *TaMIXTA1* and *TaMIXTA2* (BSMV-*TaMIXTA1as* + BSMV-*TaMIXTA2as*) (Figure 2B). In contrast, single silencing of the *TaMIXTA1* or *TaMIXTA2* gene failed to result in a significant change in cuticular wax accumulation (Figure 2B). Further cuticular wax composition analyses revealed that VLC alcohols, VLC alkanes, VLC aldehydes, and VLC esters all showed significant reduction in the wheat leaves co-silencing *TaMIXTA1* and *TaMIXTA2* compared with the BSMV-*γ* control (Figure 2C). However, the accumulation of these tested cuticular wax components was not affected by the single silencing of the *TaMIXTA1* or *TaMIXTA2* gene in wheat plants (Figure 2C). These data support that wheat genes *TaMIXTA1* and *TaMIXTA2* might act redundantly to stimulate cuticular wax accumulation.

Thereafter, excised-leaf water loss and chlorophyll leaching assays were performed to examine the potential regulation of cuticle permeability by wheat *TaMIXTA1* and *TaMIXTA2* genes. As shown in Figure 2D,E, a higher water loss rate and faster chlorophyll leaching were observed on the wheat leaves co-silencing *TaMIXTA1* and *TaMIXTA2* compared with the BSMV-*γ* control, suggesting that the cuticle permeability of wheat leaves was significantly potentiated by co-silencing *TaMIXTA1* and *TaMIXTA2*. In contrast, single silencing the wheat *TaMIXTA1* or *TaMIXTA2* genes failed to significantly affect leaf water loss and chlorophyll leaching (Figure 2D,E and Supplemental Figure S1). These results suggested that wheat genes *TaMIXTA1* and *TaMIXTA2* redundantly stimulate cuticular wax accumulation and strengthen the surface barrier property of the cuticle.

### 2.3. Identification of Wheat TaCER5 Based on Homology with Arabidopsis AtCER5

**Figure 3:**
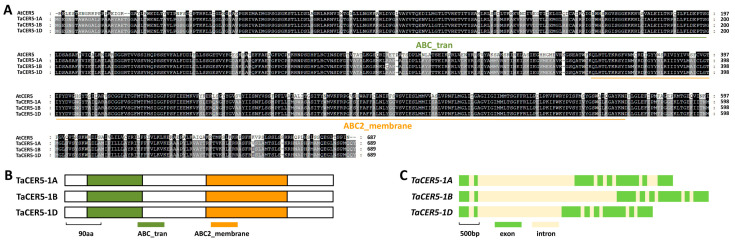
Identification of wheat TaCER5 based on homology with Arabidopsis AtCER5. (**A**) Protein sequence alignment of wheat TaCER5 and Arabidopsis AtCER5. Identical residues among 4 protein sequences are shaded in black, while residues conserved in at least 2 of the 4 proteins are shaded in gray. (**B**) Domain structures of wheat TaCER5 and Arabidopsis AtCER5. (**C**) Gene architectures of wheat *TaCER5* genes.

In *A. thaliana*, MIXTA-like transcription factors AtMYB16 and AtMYB106 could positively regulate the expression of wax deposition gene *TaCER5* and wax biosynthesis gene *TaKCS1* [74,75]. In this research, we first employed the Arabidopsis AtCER5 (At1g51500) as a query to search the reference genome of allohexaploid bread wheat. Wheat TaCER5s were identified as the closed homologs of Arabidopsis AtCER5. As shown in Figure 3A, *TaCER5-1A* (*TraesCS1A02G126900*), *TaCER5-1B* (*TraesCS1B02G147700*), and *TaCER5-1D* (*TraesCS1D02G126600*) located on chromosomes 1A, 1B, and 1D of allohexaploid bread wheat are three highly homologous sequences of the *TaCER5* gene.

Amino acid sequences of the TaCER5-1A, TaCER5-1B, and TaCER5-1D proteins shared above 62% identities with the Arabidopsis AtCER5 protein (Figure 3A). As depicted in Figure 3B, ABC transporter (ABC_tran) and ABC2-type transporter (ABC2_membrane) motifs appear in the N-terminal and C-terminal parts of TaCER5-1A, TaCER5-1B, and TaCER5-1D proteins, respectively. Gene architecture analysis revealed that eight exons and seven introns constitute the coding regions of genomic sequences of *TaCER5-1A*, *TaCER5-1B*, and *TaCER5-1D* genes (Figure 3C).

### 2.4. Wheat TaCER5 Gene Is Required for the Deposition of Cuticular Wax

**Figure 4:**
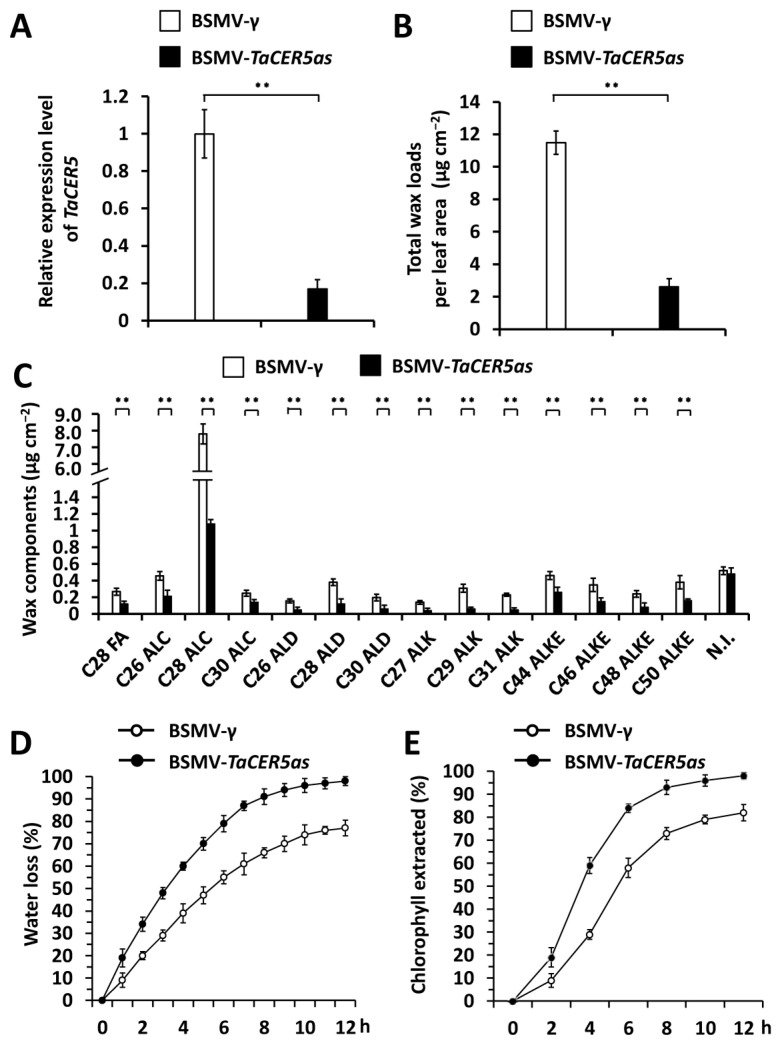
Functional analyses of wheat *TaCER5* genes in cuticular wax deposition. (**A**) qRT-PCR analysis of *TaCER5* expression levels in the leaves of wheat plants infected with BSMV-*γ* or BSMV-*TaCER5as*. (**B**) Cuticular wax loads on the leaves of wheat plants infected with BSMV-*γ* or BSMV-*TaCER5as*. (**C**) Loads of major cuticular wax components in the leaves of wheat plants infected with BSMV-*γ* and BSMV-*TaCER5as* wheat leaves. FA, fatty acid; ALC, alcohol; ALD, aldehyde; ALK, alkane; ALKE, alkyl ester; N. I., not identified compound. (**D**) Water loss rates and (**E**) chlorophyll extraction levels measured in the leaves of wheat plants infected with BSMV-*γ* or BSMV-*TaCER5as*. For (**A**–**E**), three biological replicates were statistically analyzed for each treatment, and data are presented as the mean ± SE (Student’s *t*-test, ** *p* < 0.01).

To examine the potential role of the wheat *TaCER5* gene in cuticular wax accumulation, we silenced all endogenous *TaCER5* genes by BSMV-VIGS in the leaves of the wheat cultivar Yannong 999. The qRT-PCR assay demonstrated that transcript levels of *TaCER5* genes decreased significantly in the leaves of wheat plants infected with BSMV-*TaCER5as* compared with the BSMV-*γ* control (Figure 4A). The GC-MS assay showed that the cuticular wax loads on wheat leaves decreased from 11.48 μg cm^−2^ in the BSMV-*γ* control plants to 2.62 μg cm^−2^ in the wheat plants silencing *TaCER5* (BSMV-*TaCER5as*) (Figure 4B). Further wax composition analyses revealed that loads of VLC alcohols, VLC alkanes, VLC aldehydes, and VLC esters all decreased significantly in the wheat leaves silencing *TaCER5* compared with the BSMV-*γ* control (Figure 4C). These results support that the wheat *TaCER5* gene is essential for cuticular wax deposition.

Excised-leaf water loss and chlorophyll leaching assays were then performed to examine the cuticle permeability. As shown in Figure 4D,E and Supplemental Figure S1, a higher water loss rate and chlorophyll extraction levels were observed on the wheat leaves silencing *TaCER5* compared with the BSMV-*γ* control, suggesting that cuticle permeability was reduced by silencing *TaCER5* in wheat leaves. These results collectively suggested that the wheat *TaCER5* gene is essential for cuticular wax deposition and cuticle surface barrier properties.

### 2.5. Transcriptional Activators TaMIXTA1 and TaMIXTA2 Directly Activate Transcription of TaCER5 and TaKCS1 Genes

**Figure 5:**
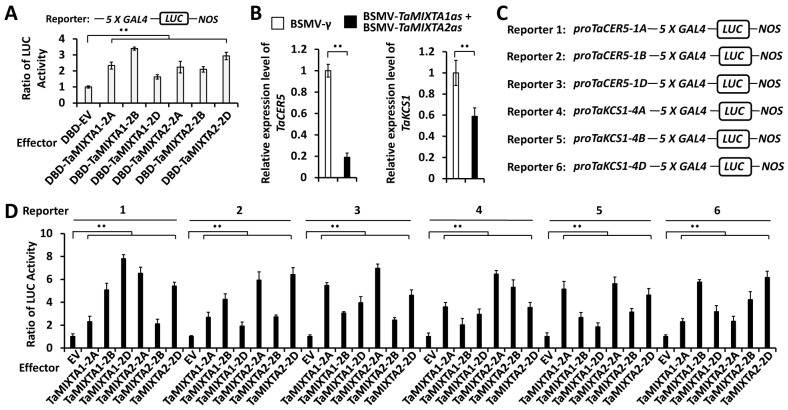
Analysis of the transcriptional activation of the *TaCER5* and *TaKCS1* genes by wheat MIXTA-like transcription factors. (**A**) Transcriptional activation activity of wheat TaMIXTA1 and TaMIXTA2 measured in Arabidopsis protoplast cells. LUC activity was normalized to that obtained from protoplasts expressing DBD alone. (**B**) Expression levels of *TaCER5* and *TaKCS1* in the wheat leaves co-silencing *TaMIXTA1* and *TaMIXTA2* were measured by qRT-PCR assay. BSMV-*γ* empty vector was employed as the negative control. (**C**) Schematic depiction of the LUCIFERASE (LUC) reporter containing promoter fragments of *TaCER5* and *TaKCS1* genes. (**D**) Activation of *TaCER5* and *TaKCS1* promoters by wheat TaMIXTA1 and TaMIXTA2 in Arabidopsis protoplast cells. LUC activity was normalized to that obtained from protoplasts expressing empty vector (EV) alone. For (**A**,**B**,**D**), three biological replicates were statistically analyzed for each treatment, and data are presented as the mean ± SE (Student’s *t*-test, ** *p* < 0.01).

Arabidopsis MIXTA-like transcription factors AtMYB16 and AtMYB106 function as transcriptional activators. In this study, we employed the dual-Luciferase reporter assay system to examine the transcriptional activation activity of the TaMIXTA1 and TaMIXTA2 proteins. As shown in Figure 5A, the expression of effectors DBD-TaMIXTA1-2A, DBD-TaMIXTA1-2B, DBD-TaMIXTA1-2D, DBD-TaMIXTA2-2A, DBD-TaMIXTA2-2B, or DBD-TaMIXTA2-2D increased the reporter luciferase activity (LucA) ratio to above 1.63 from 1 for the DBD control. This result indicates that the TaMIXTA1 and TaMIXTA2 proteins have transcriptional activation activity.

AtMYB16 and AtMYB106, Arabidopsis homologs of wheat TaMIXTA1 and TaMIXTA2 proteins, have been demonstrated to directly activate *AtCER5* and *AtKCS1* genes. To test whether wheat-redundant TaMIXTA1 and TaMIXTA2 regulate the transcription of the *TaCER5* and *TaKCS1* genes, we first analyzed the expression levels of the *TaCER5* and *TaKCS1* genes in the wheat leaves silencing the *TaMIXTA1* and *TaMIXTA2* genes. As shown in Figure 5B, the qRT-PCR assay demonstrated that expression levels of the *TaCER5* and *TaKCS1* genes were significantly reduced in wheat leaves co-silencing *TaMIXTA1* and *TaMIXTA2* compared with the control BSMV-*γ* leaves, suggesting that redundant TaMIXTA1 and TaMIXTA2 proteins positively regulate the expression of the *TaCER5* and *TaKCS1* genes.

Thereafter, we employed the dual-Luciferase reporter assay system to examine the potential transactivation of *TaCER5* and *TaKCS1* promoters by transcriptional activators TaMIXTA1 and TaMIXTA2. In the Arabidopsis leaf protoplast transfection system, LUC reporters containing promoter regions of the *TaCER5-1A*, *TaCER5-1B*, *TaCER5-1D*, *TaKCS1-4A*, *TaKCS1-4B*, and *TaKCS1-4D* genes were co-expressed with TaMIXTA1-2A, TaMIXTA1-2B, TaMIXTA1-2D, TaMIXTA2-2A, TaMIXTA2-2B, or TaMIXTA2-2D effector protein (Figure 5C). As shown in Figure 5D, the expression of effector TaMIXTA1-2A, TaMIXTA1-2B, TaMIXTA1-2D, TaMIXTA2-2A, TaMIXTA2-2B, or TaMIXTA2-2D resulted in the increase in the LucA ratio to above 1.71 from 1 for the empty vector (EV) control, suggesting that wheat TaMIXTA1 and TaMIXTA2 transcriptional activators, including TaMIXTA1-2A, TaMIXTA1-2B, TaMIXTA1-2D, TaMIXTA2-2A, TaMIXTA2-2B, and TaMIXTA2-2D, could directly activate promoters of the *TaCER5* and *TaKCS1* genes. These above results collectively support that wheat MIXTA-like transcriptional activators TaMIXTA1 and TaMIXTA2 activate the *TaCER5* and *TaKCS1* genes and stimulate cuticular wax accumulation.

## 3. Discussion

### 3.1. Wheat MIXTA-like Transcription Factors TaMIXTA1 and TaMIXTA2 Are Major Regulators of Cuticular Wax Accumulation

In response to developmental and environmental cues, plant cuticular wax biosynthesis is tightly regulated at transcriptional, post-transcriptional, post-translational, and even epigenetic levels [21]. In the dicot model plant *A. thaliana*, a plethora of regulators including transcription factors have been identified to be essential for cuticular wax accumulation [21]. For instance, AP2/ERF transcription factors AtSHN1/WIN1, AtWRI4, and AtRAP2.4 stimulate cuticular wax biosynthesis, and DEWAX negatively regulates cuticular wax accumulation [63,64,65,66,67,68,69]. MYB transcription factors AtMYB30, AtMYB94, and AtMYB96 positively regulate wax biosynthesis by activating the transcription of wax biosynthesis genes like *AtECR*, *AtKCS1*, and *AtCER1* [70,71,72,73]. Although cuticular wax accumulation in bread wheat is less understood compared with *A. thaliana*, wheat transcription factors governing wax accumulation have been increasingly identified in recent years. For instance, wheat AP2/ERF transcription factor TaWIN1/SHN1, basic helix-loop-helix (bHLH) transcription factor TaKPAB1, and MYB transcription factors TaMYB30, TaMYB31, and TaEPBM1/MYB96 function as positive regulators of cuticular wax accumulation [85,86,87,88,89,90].

Herein, wheat MIXTA-like transcription factors TaMIXTA1 and TaMIXTA2 were demonstrated to become involved in the regulation of cuticular wax accumulation. A reduced accumulation of cuticular wax was observed in wheat leaves co-silencing *TaMIXTA1* and *TaMIXTA2* but not in wheat leaves single silencing *TaMIXTA1* or *TaMIXTA2*, indicating that wheat *TaMIXTA1* and *TaMIXTA2* genes act redundantly to stimulate cuticular wax accumulation. In the dicot model plant *A. thaliana*, MIXTA-like transcription factors AtMYB16 and AtMYB106, Arabidopsis homologs of wheat TaMIXTA1 and TaMIXTA2, also positively regulate cuticular wax accumulation, suggesting that the activation of cuticular wax accumulation by MIXTA-like transcription factors might be conserved among dicots and monocots [68,73,74]. In addition to cuticular wax accumulation, other epidermal specialization events like cutin biosynthesis, trichome formation, and even stomatal development have been altered in the Arabidopsis *myb16* or *myb106* mutant [68,91,92]. Therefore, it is intriguing to examine the potential regulation of *TaMIXTA1* and *TaMIXTA2* on these relevant epidermal specialization events like cutin biosynthesis and trichome formation in bread wheat in future research.

### 3.2. Wheat TaCER5 Is a Key Component of Cuticular Wax Deposition

Previous studies in Arabidopsis revealed that ABC transporter AtCER5 plays an important role in cuticular wax deposition [62]. Reduced stem wax loads were observed in the Arabidopsis *cer5* mutants, and the amounts of all wax components such as VLC alkanes, VLC ketones, and VLC alcohols were significantly reduced on the surface of *cer5* mutants [62]. In this study, the wheat *TaCER5* gene was demonstrated to be essential for cuticular wax deposition. Total wax loads were significantly reduced on the wheat leaves silencing *TaCER5* genes, and major components, including VLC alcohols, VLC alkanes, and VLC aldehydes, accumulate much less in the cuticular wax of wheat leaves infected with BSMV-*TaCER5as*. Although major components of cuticular wax are different between cereal crop bread wheat and dicot model plant *A. thaliana*, the loss of function of *CER5* genes results in defective cuticular wax accumulation in both plant species. These studies strongly support that the contribution of *CER5* to cuticular wax deposition might be conserved among dicots and monocots.

### 3.3. Transcriptional Activators TaMIXTA1 and TaMIXTA2 Activate Transcription of TaKCS1 and TaCER5 Genes to Potentiate Wax Accumulation

Arabidopsis MIXTA-like transcription factors AtMYB16 and AtMYB106 have been demonstrated to function as transcriptional activators [68,74,75]. In this study, wheat TaMIXTA1 and TaMIXTA2 proteins, including TaMIXTA1-2A, TaMIXTA1-2B, TaMIXTA1-2D, TaMIXTA2-2A, TaMIXTA2-2B, and TaMIXTA2-2D, exhibit transcriptional activation activity, suggesting that MIXTA-like proteins AtMYB16, AtMYB106, TaMIXTA1, and TaMIXTA2 all function as transcriptional activators. Consistent with this, TaMYB16 allelic to the TaMIXTA1-2D was demonstrated to exhibit transcriptional activation ability in yeast cells [93]. In the dicot model plant *A. thaliana*, AtMYB16 and AtMYB106 could activate a plethora of cuticle biosynthesis genes such as the wax deposition gene *AtCRE5* and wax biosynthesis gene *AtKCS1* [68,74,75]. TaKCS1, a wheat homolog of Arabidopsis AtKCS1, has been recently identified as an essential component of wax biosynthesis [86]. The silencing of the wheat *TaKCS1* gene by the BSMV-VIGS experiment results in a significant reduction in cuticular wax accumulation, and all major components such as VLC alcohols, VLC alkanes, and VLC aldehydes accumulate much less in the cuticular wax of wheat leaves silencing the *TaKCS1* gene [86]. In this study, the expression levels of wax biosynthesis gene *TaKCS1* and wax deposition gene *TaCER5* were reduced in the wheat leaves co-silencing *TaMIXTA1* and *TaMIXTA2* genes. Importantly, all TaMIXTA1 and TaMIXTA2 proteins, including TaMIXTA1-2A, TaMIXTA1-2B, TaMIXTA1-2D, TaMIXTA2-2A, TaMIXTA2-2B, and TaMIXTA2-2D, could activate promoters of the *TaCER5* and *TaKCS1* genes, supporting that transcriptional activators TaMIXTA1 and TaMIXTA2 could directly activate the transcription of the *TaKCS1* and *TaCER5* genes. These studies suggest that the transcriptional activation of wax biosynthesis gene *TaKCS1* and wax deposition gene *TaCER5* by MIXTA-like transcription factors might be conserved in cereal crop bread wheat and dicot model plant *A. thaliana*.

Up to now, a variety of wheat transcription factors such as TaWIN1/SHN1, TaMYB30, TaEPBM1/MYB96, TaMYB31, TaMIXTA1, and TaMIXTA2 stimulating cuticular wax accumulation have been identified [85,86,87,88,89,90]. Arabidopsis AP2/ERF transcription factor DEWAX negatively regulates cuticular wax accumulation, but wheat transcription factors suppressing cuticular wax accumulation remain unknown [76,77,78]. Furthermore, increasing evidence revealed that cuticular wax accumulation is tightly regulated by developmental and environmental cues. For instance, Arabidopsis transcription factor AtMYB96 functions to activate cuticular wax biosynthesis under drought stress [73]. Therefore, it is intriguing to examine the potential regulation of wheat transcription factors like TaMIXTA1 and TaMIXTA2 on the response of cuticular wax biosynthesis to developmental and environmental cues in future research. In addition, wheat wax biosynthesis genes directly targeted by transcription factors TaMYB30, TaEPBM1/MYB96, TaMIXTA1, and TaMIXTA2 have been characterized so far [86,90]. For instance, the wax biosynthesis gene *TaECR* was activated by transcription factors TaMYB30 and TaEPBM1/MYB96, and the *TaKCS1* gene was targeted by TaMYB30, TaMIXTA1, and TaMIXTA2, suggesting that one wax biosynthesis gene could be regulated by more than one transcription factor [86,90]. Characterizing genome-wide binding sites and target genes of these transcription factors would certainly contribute to our understanding of wheat cuticular wax accumulation at the transcriptional level.

## 4. Materials and Methods

### 4.1. Plant Materials

Wheat cultivar Yannong 999 and *A. thaliana* ecotype Columbia (Col-0) were employed as plant materials in this research. Wheat cultivar Yannong 999 was used for qRT-PCR, BSMV-VIGS, wax composition analysis, water loss, and chlorophyll leaching assays, whereas *A. thaliana* Col-0 was employed for transcriptional activation analysis. After surface disinfection as previously described [94], wheat seeds were planted in 300 mL pots containing an autoclaved soil mixture of horticultural compost, sand, and silt-loam soil (1:1:2 *v*/*v*/*v*), and they were grown in climate chambers under 16 h light/8 h dark, 20 °C/18 °C day/night cycle, and 70% relative humidity (RH). The plants were irrigated three times per week with 60 mL of distilled water per pot. After surface sterilization, Arabidopsis seeds were planted in 200 mL pots containing a commercial soil mix (Pindstrup, Ryomgaard, Denmark) and grown in climate chambers at 22 °C and 70% RH under a 16 h light/8 h dark photoperiod. The plants were irrigated three times per week with 60 mL of distilled water per pot.

### 4.2. Protein Alignment and Domain Analysis

The protein sequences were subjected to alignment with the MegAlign program by the Clustal W method. The protein domains were identified from the Interpro Pfam database [95].

### 4.3. qRT-PCR Assay

The qRT-PCR assays analyzing gene expression levels of *TaMIXTA1*, *TaMIXTA2*, *TaCER5*, and *TaKCS1* in BSMV-VIGS wheat leaves were conducted as previously described [86]. Total RNA was extracted using the TRIzol Reagent (Invitrogen, Carlsbad, CA, USA). The RNA quality was examined according to previous studies [96,97]. An amount of 2 μg of total RNA was used to generate the cDNA template under the TransScript one-step gDNA removal and cDNA synthesis supermix (Transgenbiotech, Beijing, China) according to the manufacturer’s instructions. The cDNA was used as a template in the subsequent real-time PCR assay performed under the ABI real-time PCR system with the qPCR Master Mix (Invitrogen, Carlsbad, CA, USA). The expression levels of *TaMIXTA1*, *TaMIXTA2*, *TaCER5*, and *TaKCS1* were measured by using primers listed in Supplemental Table S1. These primers used for the *qRT-PCR* assay were designed by the primer premier 5 design program and the primer efficiency was analyzed as previously described [98].

### 4.4. BSMV-VIGS Assay

The BSMV-VIGS assays silencing *TaCER5*, *TaMIXTA1*, *TaMIXTA2*, or co-silencing *TaMIXTA1* and *TaMIXTA2* were conducted as previously described by Liu et al. [86]. Fragments of *TaCER5*, *TaMIXTA1*, and *TaMIXTA2* were amplified using the primers listed in Supplemental Table S1, and PCR products were employed for generating constructs of the vectors BSMV-*TaCER5as*, BSMV-*TaMIXTA1as*, and BSMV-*TaMIXTA2as*.

### 4.5. Cuticular Wax Composition Analysis

The wax constituents in the cuticle of BSMV-VIGS wheat leaves were measured as previously described [86]. Briefly, cuticular wax mixtures of wheat leaves were extracted with chloroform (Merck, Rahway, NJ, USA) by dipping wheat leaves into this organic solvent. After being dried under N_2_ gas, extracts were analyzed by using a capillary GC (5890 Series II, Agilent Technologies, Santa Clara, CA, USA) and a flame ionization detector (6890 N, Agilent Technologies) with a mass spectrometer (MSD 5973, Agilent Technologies). Details of the oven temperature program were set as described [86]. Wax components were identified based on retention times compared to known standards and quantified based on flame ionization detector peak areas compared to the internal standard.

### 4.6. Water Loss and Chlorophyll Leaching Assay 

The water loss and chlorophyll leaching assays analyzing cuticle permeability of BSMV-VIGS wheat leaves were conducted as previously described [86]. Briefly, wheat plants were dipped in ultrapure water for 1 h in the dark to maintain stomatal closure, and the leaves were detached. For the water loss rate tests, the weights of detached leaves were then measured each hour for 12 h. For the chlorophyll leaching assay, chlorophyll was extracted from detached leaves with 80% ethanol and measured with a spectrophotometer each hour for 12 h. Total leaf chlorophyll was extracted using DMSO in a 65 °C incubator in the dark. After the addition of 80% (*v*/*v*) acetone, total chlorophyll content was measured and normalized with the total chlorophyll content measured in leaves from BSMV-γ infected plants (control).

### 4.7. Transcriptional Activation Analysis

The transcriptional activation analysis measuring the transactivation activity of transcription factors TaMIXTA1 and TaMIXTA2 in *Arabidopsis* protoplast cells was conducted using the dual-Luciferase reporter assay system according to the manual. The preparation and transformation of the *Arabidopsis* protoplast were conducted as described previously by Zhi et al. [99]. Arabidopsis mesophyll protoplasts were transfected with indicated reporter and effector constructs. The reporter luciferase activity (LucA) was analyzed about 48 h after protoplast transfection, and the Gal4 DNA-binding domain (DBD) was used to determine the basal LUC activity.

## 5. Conclusions

Herein, wheat MIXTA-like transcription factors TaMIXTA1 and TaMIXTA2 were characterized as positive regulators of cuticular wax accumulation. Knock-down of wheat *TaMIXTA1* and *TaMIXTA2* expressions by virus-induced gene silencing resulted in the decreased accumulation of leaf cuticular wax and increased leaf cuticle permeability. Furthermore, TaCER5, the wheat homolog of Arabidopsis ABC transporter AtCER5, was identified as a key component of cuticular wax deposition. The silencing of *TaCER5* by BSMV-VIGS led to reduced cuticular wax loads and enhanced rates of leaf water loss and chlorophyll leaching. Importantly, we demonstrated that TaMIXTA1 and TaMIXTA2 function as transcriptional activators and could directly stimulate the transcription of wax biosynthesis gene *TaKCS1* and wax deposition gene *TaCER5*. These results strongly support that wheat MIXTA-like transcriptional activators TaMIXTA1 and TaMIXTA2 positively regulate cuticular wax accumulation via activating *TaKCS1* and *TaCER5* gene transcription. These findings could expand our knowledge of wheat cuticular wax accumulation and contribute to molecular breeding for wheat’s resistance against environmental stresses like drought.

## Data Availability

Data presented here are available on request by correspondence.

## Supplementary Materials

The following supporting information can be downloaded at https://www.mdpi.com/article/10.3390/ijms25126557/s1.
