# Supplementary material for: Wheat MIXTA-like Transcriptional Activators Positively Regulate Cuticular Wax Accumulation
Source: Int J Mol Sci. 2024 Jun 14;25(12):6557. doi: 10.3390/ijms25126557 (PMC11204111; doi:10.3390/ijms25126557)
Supplement: Supplementary file 1 [file ijms-25-06557-s001.zip › ijms-3032127-supplementary.pdf]

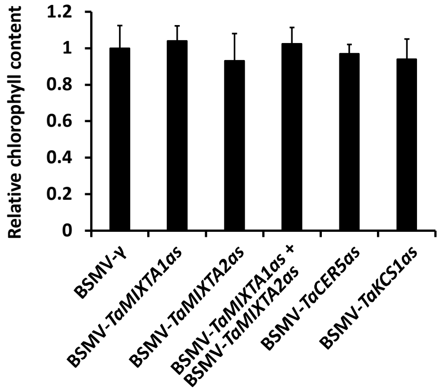

Figure S1 Chlorophyll content in BSMV-VIGS wheat plants.

Table S1. Primers used in this study

Primer Name	Sequence	Annotation
qRT-PCR-*TaMIXTA1*-F	5' CAAGCCGCGCTCCGACGTG3'	qRT-PCR primer for *TaMIXTA1*, F primer
qRT-PCR-*TaMIXTA1*-R	5' GTGGACGTCGGCGAGTCGA3'	qRT-PCR primer for *TaMIXTA1*, R primer
qRT-PCR-*TaMIXTA2*-F	5' CAAGGCCACCAACGGAGCT3'	qRT-PCR primer for *TaMIXTA2*, F primer
qRT-PCR-*TaMIXTA2*-R	5' GTGGAGGTGGGTGACTCCA3'	qRT-PCR primer for *TaMIXTA2*, R primer
qRT-PCR-*TaCER5*-F	5' AGCTTGACATGGGAGAAC3'	qRT-PCR primer for *TaCER5*, F primer
qRT-PCR-*TaCER5*-R	5' GCCTCTTCTTGCCGTTGA3'	qRT-PCR primer for *TaCER5*, R primer
qRT-PCR-*TaKCS1*-F	5' CTACTCCTTCGTCCGCCTC3'	qRT-PCR primer for *TaKCS1*, F primer
qRT-PCR-*TaKCS1*-R	5' GTGATCTTGGTCTGGAACG3'	qRT-PCR primer for *TaKCS1*, R primer
pCa-*TaMIXTA1as*-F	5'AAGGAAGTTAGTTGACCAGGTTCAGTAT 3'	For construct of BSMV-*TaMIXTA1as*, F primer
pCa-*TaMIXTA1as*-R	5'AACCACCACCACCGTGCAGCCCATGCAGGCGTA 3'	For construct of BSMV-*TaMIXTA1as*, R primer
pCa-*TaMIXTA2as*-F	5'AAGGAAGTTACCACCATGCCGAGGATGC 3'	For construct of BSMV-*TaMIXTA2as*, F primer
pCa-*TaMIXTA2as*-R	5'AACCACCACCACCGACCTGGAGTCACCCACCTC 3'	For construct of BSMV-*TaMIXTA2as*, R primer
pCa-*TaCER5as*-F	5'AAGGAAGTTGTTGAGCCCCTCCTGCATG3'	For construct of BSMV-*TaCER5as*, F primer
pCa-*TaCER5as*-R	5'AACCACCACCACCGGAGTTCATCATCACCAAC3'	For construct of BSMV-*TaCER5as*, R primer
